# Fabrication and Hydrodynamic Characterization of a Microfluidic Device for Cell Adhesion Tests in Polymeric Surfaces

**DOI:** 10.3390/mi10050303

**Published:** 2019-05-05

**Authors:** J. Ponmozhi, J. M. R. Moreira, F. J. Mergulhão, J. B. L. M. Campos, J. M. Miranda

**Affiliations:** 1 Transport Phenomena Research Center (CEFT), Department of Chemical Engineering, Faculty of Engineering, University of Porto, Rua Dr. Roberto Frias s/n, 4200-465 Porto, Portugal; jponmozhi@gmail.com (J.P.); jmc@fe.up.pt (J.B.L.M.C.); 2 Laboratory for Process Engineering, Environment (LEPABE), Biotechnology and Energy, Department of Chemical Engineering, Faculty of Engineering, University of Porto, Rua Dr. Roberto Frias s/n, 4200-465 Porto, Portugal; joanarm@fe.up.pt (J.M.R.M.); filipem@fe.up.pt (F.J.M.)

**Keywords:** cell adhesion, biomedical coatings, microfabrication, computational fluid dynamics, microfluidics

## Abstract

A fabrication method is developed to produce a microfluidic device to test cell adhesion to polymeric materials. The process is able to produce channels with walls of any spin coatable polymer. The method is a modification of the existing poly-dimethylsiloxane soft lithography method and, therefore, it is compatible with sealing methods and equipment of most microfluidic laboratories. The molds are produced by xurography, simplifying the fabrication in laboratories without sophisticated equipment for photolithography. The fabrication method is tested by determining the effective differences in bacterial adhesion in five different materials. These materials have different surface hydrophobicities and charges. The major drawback of the method is the location of the region of interest in a lowered surface. It is demonstrated by bacterial adhesion experiments that this drawback has a negligible effect on adhesion. The flow in the device was characterized by computational fluid dynamics and it was shown that shear stress in the region of interest can be calculated by numerical methods and by an analytical equation for rectangular channels. The device is therefore validated for adhesion tests.

## 1. Introduction

The term ‘biofilm’ was coined in 1981 [1] and refers to a community of microbial cells that is attached to a surface and enclosed in a self-produced exopolymeric matrix mainly composed by polysaccharide material [2]. Biofilm formation is due to the onset of adhesion of cells to a surface. The development of biofilms in medical devices is a common problem, which can lead to hospitalization, revision surgery due to microbiological implant colonization or mortality.

Recent reviews provide a source of evidence to the undesirable biofilm formation in medical devices [3,4,5,6,7,8,9]. These biofilms pose a challenge to the health care community. Currently, to reduce biofouling, there is a spurring development of smart polymers that are used for coating biomedical implants, artificial organs, lab on chip surfaces, implantable drug delivery systems [10], giving way to the development of next generation biomedical devices with reduced fouling.

Many types of measures were proposed to combat biofilms, like conventional usage of antibiotics or surfaces designed to inhibit initial adhesion of cells, for the first stage of biofilm formation [11]. A clear understanding of the initial adhesion mechanisms can lead to the development of ideal biomaterials in which cells are unable to attach and growth of biofilms would be hindered [12]. Consequently, there arises a need to quantify and understand the initial adhesion phenomenon over diverse materials.

Different types of biomaterials were used throughout the past 30 years, for different types of biomedical applications. Ramakrishna, et al. [13] have given a brief introduction of different biocompatible polymers used for different types of implants. For example, poly-l-lactide acid (PLLA) is a biodegradable polymer, which degrades over time without harmful consequences to the body [14,15]. Different types of pins and screws, coated with PLLA, are used to fix the implants. Autografts, namely suspensory fixation, hamstring fixation [16] are used as fixing agents for femoral implants to reduce operation failures.

Recently, researchers started taking advantage of microfluidic devices for biofilm research [17,18,19] due to several benefits:

- The different biological cells can be monitored in real time with microscopy visualization techniques;
- Low-cost assays and reagents can be used in the microliter range helping in cost cutting;
- Leak-proof inlet and outlet connections can be made easily as the poly-dimethylsiloxane (PDMS) microfluidic channels are deformable;
- The surface can be easily modified and the geometry can be designed according to the application;
- The flow is always laminar, even at a high shear stress range.

In the particular case of adhesion tests, tests can be conducted in a simpler and smaller setup. The pumps required have lower power, the amount of fluid is smaller, and the setup is more flexible. Multiple tests can be conducted in parallel in a single chip and new geometries are easy to produce. Additionally, by using microfluidic devices, researchers can easily mimic the dynamical conditions of biomedical settings. It has been shown that adhesion is influenced by the flow patterns near the wall, the wall shear stress being the most important parameter used to characterize the flow influence on adhesion. For this reason, microdevices to study the wall adhesion must have well characterized hydrodynamic conditions. It is crucial that the wall shear stress is predictable given the flow rate used in the experiments. Additionally, geometrical features of the device should not interfere with the flow or with adhesion in the regions of interest. For this reason, straight channels are the preferred configuration for dynamic adhesion tests. New cells need to be validated to assure they are adequate for routine adhesion tests. Computational fluid dynamics (CFD) is usually used to characterize the flow and calculate the wall shear stress.

To aid the vast developing applications based on microfluidics, there are many conventional rapid prototyping techniques developed for microchannels fabrication. Duffy, et al. [20] demonstrated a method to produce PDMS channels for microfluidic applications. First, the channel is designed in Computer-Aided Design (CAD) software. The design is printed in a transparency and used as a mask for making a positive relief master mold. The master mold is produced in SU-8 polymer epoxy photoresist, step described in detail by Blanco, et al. [21] and Che-Hsin, et al. [22]. The PDMS channels are then casted with this mold and baked to obtain irreversibly sealed channels. Other conventional methods are commercially used for micromolding such as: micromilling [23], micropowder blasting [24], hot embossing [25], laser ablation [26] and stereo lithography [27].

Xurography is a technique developed by Bartholomeusz, et al. [28] utilizing a simple cutter plotter. The microchannels can be cut, according to the application, on vinyl films or other types of films. Positive relief molds, of thicknesses ranging from 25 to 1000 μm, can be generated in the cutter plotter and made ready for casting microchannels in less than 30 min. These molds can also be used as normal molds to produce PDMS microchannels through soft lithographic technique [29].

The foremost advantages of xurography technique are the reduced capital cost and manufacture time. The alternative to xurography is photolithography, a technique that needs expensive machines and clean rooms. Design modifications using photolithography techniques require more than a day, with procedures with long pre and post bake steps. The main disadvantage of xurography is the low resolution that precludes the production of microchannels with dimensions smaller than 200 μm.

Microfabrication techniques that increase the range of possible materials to be used in microfluidics adhesion tests would contribute significantly to the research progress. The objective of adhesion tests is to evaluate different materials in their original form, and therefore a fabrication procedure that changes the material must be avoided. The new techniques must be compatible with existing equipment and with microfabrication techniques, the PDMS soft lithography being the most used. In PDMS soft lithography, irreversible PDMS-PDMS sealing has some important advantages: the bond between the device and the cover is strong and there is no need to use plasma oxygen that would change the surface properties being tested. With existing techniques, PDMS does not bond easily with any polymer unless a surface treatment is applied that would change the surface properties of the polymer. The techniques must also be inexpensive and with short development cycles.

In this work we introduce a technique to incorporate a small wall patch into a PDMS microchannel produced from molds easily made in-house by xurography. The molds can be produced through any other technique, but xurography has low costs and large accessibility. The wall patch can be made of different polymers. The technique is useful to produce channels for adhesion tests, by adapting a standard PDMS soft-lithography. The procedure is compatible with irreversible PDMS-PDMS sealing and does not require plasma oxygen or any other surface treatment. Care was taken to validate the device for adhesion tests performing adhesion experiments and flow numerical simulations. Since one of the key factors influencing cell adhesion is the wall shear stress, the flow in the device was characterized by computational fluid dynamics and the wall shear stress was calculated for the conditions studied.

## 2. Materials and Methods

### 2.1. Mold Preparation

The molds of the microchannels were produced by xurography [28], a technique that uses a cutter-plotter to produce molds by removing excess material from adhesive films. The design of the models was made in CorelDRAW. Afterwards, the design was copied to GreatCut software, which instructed an Expert24 GCC plotter (GCC, New Taipei City, Taiwan) to cut the microchannels.

Four different polymer films were tested to check compatibility with PDMS soft lithography [29]. Adhesive film obtained from Sadipal (Girona, Spain) showed to be the most suitable and was selected for mold production. The thickness of the Sadipal adhesive films used was 100 μm and this was the height of the microchannel.

### 2.2. PDMS Soft Lithography

Microchannels were made from poly-dimethylsiloxane (PDMS) using soft lithography techniques [29]. The microchannels were prepared with a homogenous mixture of PDMS and curing agent (Sylgard 184, Dow Corning, Midland, MI, USA) at a ratio of 5:1. A desiccator connected to the vacuum pump was used to remove the air bubbles formed during the PDMS mixing process. The PDMS mixture was poured over a mold and kept in the oven for 20 min at 80 °C. After curing, the PDMS microchannel was peeled off from the mold. Holes of 1 mm in diameter were punched through the PDMS replicas, at both ends of the channel, to provide inlet and outlet flow with the help of a syringe tip. The PDMS microchannels were sealed with a PDMS coated thin slab (usually a glass slide, see next section) and kept in the oven for approximately 12 h at 80 °C. The sealing method is based on partial curing PDMS-PDMS bonding without plasma treatment described in the literature [30].

### 2.3. Insertion of Polymer Wall Patches in the Channels

To test the adhesion of cells in a given material, a patch of the material must be inserted in one of the walls of the channel (usually the bottom wall). With this in mind, channels comprising 5 different wall materials were fabricated by modifying the sealing slides. In one case, polystyrene cover was used as substrate to fabricate microchannels with polystyrene wall surfaces, while in the other 4 cases different polymers were spincoated over the sealing glass slide to insert a patch of polymer in the wall of the channel.

**Figure 1:**
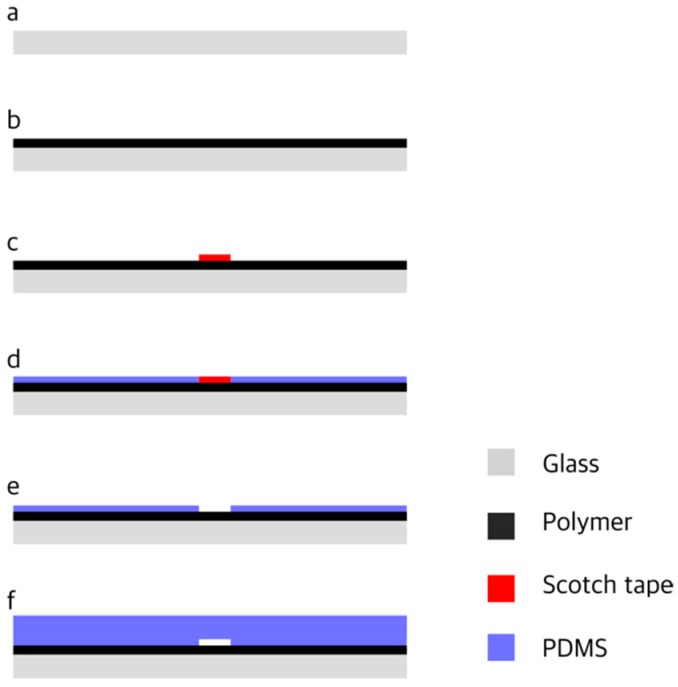
Fabrication procedure: (**a**) Substrate; (**b**) Polymer coating; (**c**) Scotch tape pasted over the polymer layer; (**d**) poly-dimethylsiloxane (PDMS) coating; (**e**) Removal of the Scotch tape; (**f**) Sealing with PDMS slab with the microchannel imprinted in it.

The coated glass slides, used to seal the channels, were prepared by a two-layer spin coating technique (Figure 1) using a WS-650S-6NPP-Lite Laurell Technologies spin coater (North Wales, PA, USA). Different volatile polymers and solvents were mixed in appropriate volume percentages (Table 1). The polymer solution was spincoated over the substrate (Figure 1b). After the formation of a polymeric film, by evaporation of the solvent, a scotch tape was pasted over the polymer surface (Figure 1c) and the PDMS was spincoated for 50 s at 5000 rpm over both the polymer and the scotch tape (Figure 1d). The scotch tape was carefully peeled off (Figure 1e) and the slide was baked for 5 min in the oven at 80 °C. A lowered surface (3 × 3 mm^2^) was left on the slide. The level of the lowered surface was determined from the experimental relation between thickness, coating speed and time [31,32] and found to be approximately 10 μm. The PDMS slab with the channel imprinted on it was sealed over the slide. The PDMS slab and the slide were aligned to ensure that the microchannel crossed the lowered surface.

The procedure can also be used to test the adhesion to the substrate (e.g., polystyrene surfaces) and in this case the first layer spin coating step (Figure 1b) is skipped. This alternative procedure can be used to produce microchannels with other surfaces, such as glass, provided that a transparent thin slab is available.

**Figure 2:**
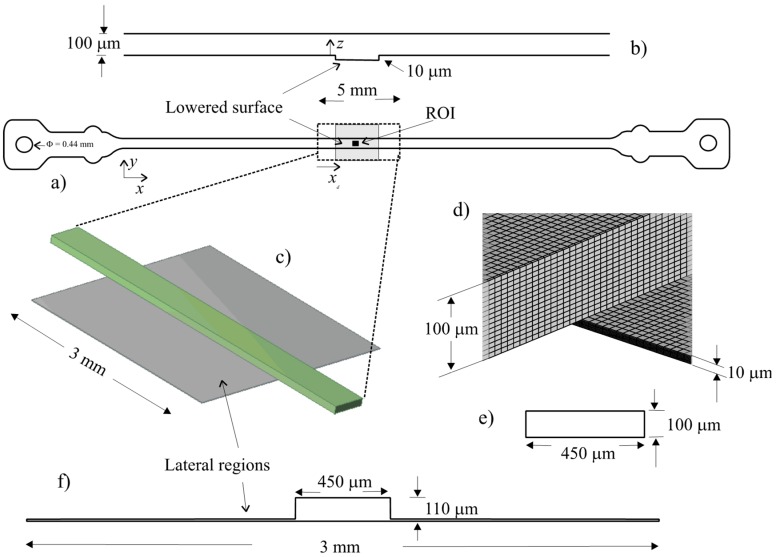
Microchannel representation and mesh details: (**a**) Microchannel, showing the lowered surface in grey, the region of interest in black and domain limits; (**b**) Profile representing the level of the upper and lower surfaces of the channel; (**c**) 3D representation of the numerical domain; (**d**) Lowered surface detail; (**e**) Microchannel cross-section outside the lowered region; (**f**) Cross-section available to the flow in the lowered surface region.

The method described above produces microchannels with PDMS surface along most of its length and a small patch of a different material located in a lowered surface half-length from the inlet (see Figure 2). The lowered surface is an unavoidable side effect of the method. To allow for PDMS-PDMS bonding, a layer of PDMS must be added above the polymer layer, with the exception of a small region that is not covered with PDMS and corresponds to the lowered surface of polymer. To test the effect of the lowered surface on bacterial cell adhesion, microchannels with PDMS walls along the full length of the microchannel were produced following the double layer technique, in which both layers are made of PDMS.

### 2.4. Bacteria and Culture Conditions

*Escherichia coli* JM109(DE3) was used, since this strain had already demonstrated a good adhesion capacity [33]. A starter culture was prepared as described by Teodosio, et al. [34] and incubated overnight. A volume of 60 mL from this culture was centrifuged (for 10 min at 3202× *g*) and the cells were washed twice with citrate buffer 0.05 M [35], pH 5.0. The pellet was then resuspended and diluted in the same buffer to obtain a cell concentration of 7.6 × 10^7^ cell mL^−1^.

### 2.5. Cell Adhesion Test

Cell adhesion tests with *E. coli* were performed with different polymer channels. Three trials were conducted for each material. The cell suspension was pumped using a syringe pump (Cetoni, neMESYS syringe pump, Korbussen, Germany) through a tygon^®^ tube to the microfluidic channel. Adhesion was followed using a fluorescence inverted microscope (DMI 5000M, Leica Microsystems GmbH, Wetzlar, Germany) with a 40× objective. Microscopic images were captured with a CCD camera (Leica DFC350FX, Leica Microsystems GmbH, Wetzlar, Germany) with a time interval of 60 s. The image sequence obtained is in tiff format as recorded by Leica Application Suite software. The area of the region of interest was 312 × 233 μm^2^. Initial adhesion experiments lasted for 1800 s.

### 2.6. Flow Conditions

The flow conditions studied are indicated in Table 2. With these conditions, the shear stress range is from 0.01 Pa to 1 Pa, covering the majority of shear stresses that can be found in the human body [36,37].

### 2.7. Image Analysis

All the 30 images were imported to Image J software [38]. A low noise region was selected in the images using the crop tool. The images were converted from 8 bit to 32 bit to improve contrast. To set the scale for processing, the pixel aspect ratio was set to one. Depending on the images, they can be smoothed using the mean filter with 1 pixel as radius. Then, the background was subtracted with rolling ball radius ranging from 1 to 18 pixels. A light background was obtained with the *E. coli* cells bright and visible. The brightness and contrast were fine-tuned to get more accurate cell count. The threshold was adjusted, for the stack of images, to generate a black and white image containing black cells over a white background. Cells have a characteristic size range (from about 0.5 μm to 3 μm) and particles and noise outside the size range were filtered out. Then the cells were automatically counted. The image for *t* = 0 already shows some cells, since the first image is taken some minutes after the flow starts. The number of cells for *t* = 0 was subtract from the total of cells counted.

By observation of the images obtained it was possible to distinguish cells from other particles due the characteristic shape of *E. coli* and the progressive increase of the number of cells throughout the experiments. The automatic counting method was compared with manual counting and this comparison showed that the automatic counting was accurate.

### 2.8. Hydrophobicity Test

The surface hydrophobicity can be determined by the contact angle formed between a surface and a polar and apolar liquid drop [39]. In this work, the contact angles were determined automatically by the sessile drop method in a contact angle meter (OCA 15 Plus; Dataphysics, Filderstadt, Germany) using water, formamide and α-bromonaphtalene (Sigma-Aldrich Corporation, St. Louis, MI, USA) as reference liquids [40]. For each surface, at least 10 measurements with each liquid were performed at 25 ± 2 °C.

According to van Oss [39], the total surface energy ($\gamma^{\mathrm{TOT}}$) of a pure substance is the sum of the apolar Lifshitz-van der Waals components of the surface free energy ($\gamma^{\mathrm{LW}}$) with the polar Lewis acid-base component ($\gamma^{\mathrm{AB}}$):

(1) $$\gamma^{\mathrm{TOT}}=\gamma^{\mathrm{LW}}+\gamma^{\mathrm{AB}}$$

The polar AB component comprises the electron acceptor $\gamma^{+}$ and electron donor $\gamma^{-}$ parameters, and is given by:

(2) $$\gamma^{\mathrm{AB}}=2\sqrt{\gamma^{+}\gamma^{-}}$$

The surface energy components of a solid surface (s) are obtained by measuring the contact angles (*θ*) with the three different liquids (l), with known surface tension components, followed by the simultaneous resolution of three equations of the type:

(3) $$\left(1+\cos\theta \right)\gamma_{1}=2\left(\sqrt{\gamma_{s}^{\mathrm{LW}}\gamma_{1}^{\mathrm{LW}}}+\sqrt{\gamma_{s}^{+}\gamma_{1}^{-}}+\sqrt{\gamma_{s}^{-}\gamma_{1}^{+}}\right)$$

The degree of surface hydrophobicity is expressed as the free energy of interaction (ΔG mJ·m^−2^) between two entities of that surface immersed in a polar liquid (such as water (w) as a reference solvent). ΔG was calculated from the surface tension components of the interacting entities, using the equation:

(4) $$\Delta G=-2{\left(\sqrt{\gamma_{s}^{\mathrm{LW}}}-\sqrt{\gamma_{w}^{\mathrm{LW}}}\right)}^{2}+4\left(\sqrt{\gamma_{s}^{+}\gamma_{w}^{-}}+\sqrt{\gamma_{s}^{-}\gamma_{w}^{+}}-\sqrt{\gamma_{s}^{+}\gamma_{s}^{-}}-\sqrt{\gamma_{w}^{+}\gamma_{w}^{-}}\right)$$

If the interaction between the two entities is stronger than the interaction of each entity with water, ΔG < 0, the material is hydrophobic and if ΔG > 0, the material is hydrophilic.

Additionally, the surface charge of each polymer was characterized through the zeta potential. Particle suspensions of each material [41] were prepared in order to measure the electrophoretic mobility, using a Nano Zetasizer (Malvern Instruments, Malvern, Worcestershire, UK).

### 2.9. Numerical Simulations

The flow in the cell was simulated by numerical methods to clarify the stability and the predictability of the flow patterns near the observation region. The microchannel used has a rectangular cross section of 450 × 100 μm and a length of 15 mm. The channel is represented in Figure 2a. In Figure 2a it is possible to observe the lowered surface represented in grey, the region of interest in black and the limits of the numerical domain. The inlet and outlet have a diameter (*D*_in_) of 0.44 mm (represented in Figure 2a). A profile along the channel, showing the level of the lower and upper walls, is represented in Figure 2b.

A section of the microchannel, around the visualization region, was selected for simulation domain (Figure 2c). This region includes the lowered surface, which results from the fabrication method, where the region of interest is located. The length of the domain is 5 mm. The lowered surface has a length of 3 mm and a width of 3 mm. The lowered surface level is approximately 10 μm. The cross section available for flow in the lowered surface region is shown in Figure 2f. Outside the lowered region the cross section available for the flow is rectangular (Figure 2e). A detail of the mesh used is shown in Figure 2d.

The flow regime was determined by the Reynolds number based on the equivalent diameter of the channel:

(5) $$Re=\frac{2\rho Q}{\left(W+H\right)\mu }$$

where ρ and μ are the density and viscosity of the fluid, respectively, Q the flow rate, W the width of the channel and H the depth of the channel.

Numerical values of the wall shear stress (WSS) are compared with data from the analytical solution for the flow in a parallel plate channel [42]:

(6) $$\tau =\mu \frac{3Q}{2{\left(\frac{H}{2}\right)}^{2}W}$$

Nominal wall shear stress, used to distinguish the experiments, was calculated through the analytical Equation (6). The real wall shear stress, as given by the numerical simulation, is slightly different.

The equation for the lowered surface can be corrected by the following equation:

(7) $$\tau_{ls}=\tau {\left(\frac{H}{H_{ls}}\right)}^{2}$$

where τ is the wall shear stress in the straight channel (outside the lowered surface) and $H_{ls}$ the depth of the channel in the lowered surface section.

Numerical simulations were made with the commercial code Ansys Fluent CFD package (version 14.5) by solving Navier–Stokes equations. A model of the microchannel was built in Design Modeler 14.5 and was discretized into a grid of 278,000 cells by Meshing 14.5. The QUICK scheme [43] was used for the discretization of the momentum equations and the PRESTO! scheme for the discretization of the pressure terms. The velocity–pressure coupled equations were solved by the PISO algorithm [44]. The no slip boundary condition was considered for all the walls. Simulations were made in steady state mode until convergence. The properties of water (density and viscosity) at 37 °C were used.

Corrected numerical results were calculated by applying Equation (7). The value of τ used was the wall shear stress of a straight channel (without a lowered surface) previously obtained numerically.

## 3. Results

### 3.1. Hydrophobicity

**Figure 3:**
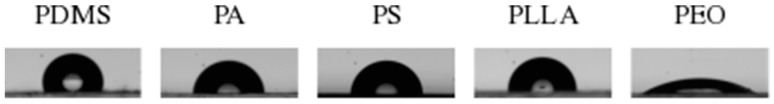
Water droplet over the surfaces of PDMS, PA, PS, poly-l-lactide acid (PLLA) and Polyethylene oxide (PEO) illustrating the hydrophobicity of the materials.

The surface properties of the different materials fabricated are presented in Table 3. Results showed that PLLA, PDMS, PA and PS are hydrophobic surfaces (ΔG < 0 mJ·m^−2^) whereas PEO is hydrophilic (ΔG > 0 mJ·m^−2^). For illustration, water droplets are shown in Figure 3 for different materials. Additionally, the zeta potential results showed that all the polymers’ surfaces have a negative charge. The range of surface characteristics assures that the procedure can be used to study a large range of surface parameters, a very important factor for bacterial adhesion studies, wherein it is desirable to cover the characteristics of all available materials used in medical or industrial applications.

### 3.2. Adhesion

**Figure 4:**
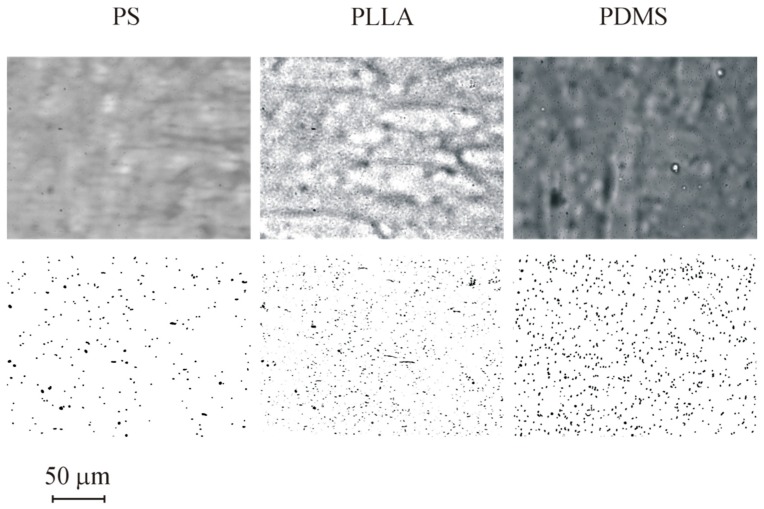
Images for adhesion in different materials for *t* = 1800 s: PS, PLLA and PDMS. Top row is before and bottom row after image processing. Results obtained for nominal wall shear stress of 0.02 Pa.

**Figure 5:**
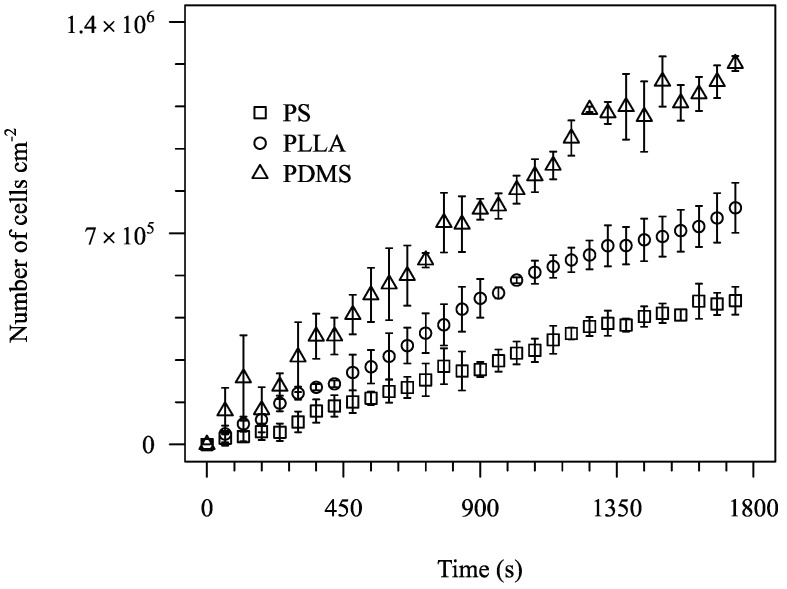
Number of adhered cells per cm^2^ for PS, PLLA and PDMS for nominal wall shear stress of 0.02 Pa.

**Figure 6:**
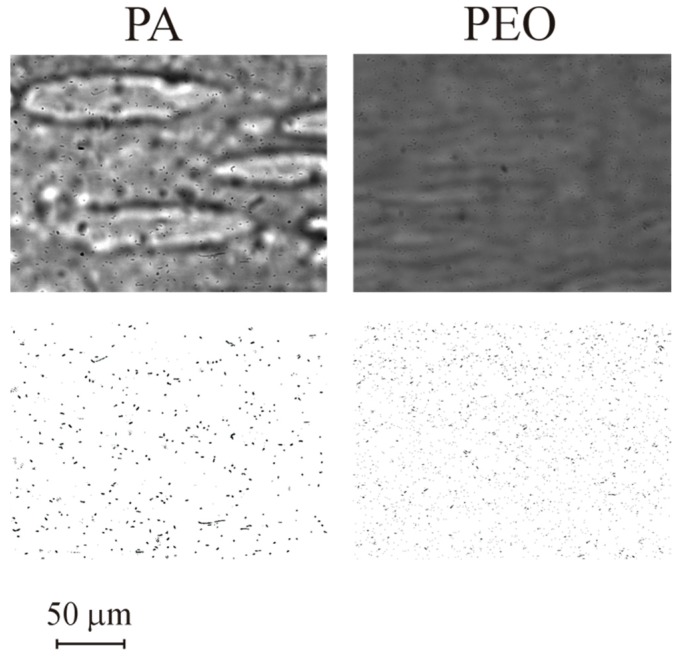
Images for adhesion in different materials for *t* = 1800 s: PA and PEO. Top row is before and bottom row after image processing. Results obtained for nominal wall shear stress of 0.01 Pa.

**Figure 7:**
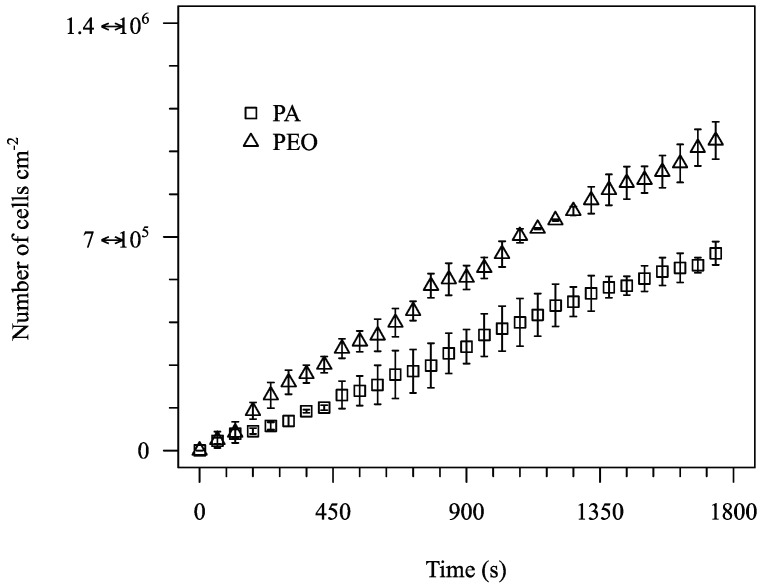
Number of adhered cells per cm^2^ for PA and PEO for nominal wall shear stress of 0.01 Pa.

Adhesion tests were performed for all 5 materials cited in Table 3. A subset of the results obtained (for PS, PLLA and PDMS) is represented in Figure 4. The images show the surface after 1800 s assays and the processed images. Cells are visible in all three surfaces studied and so the images can be processed to obtain a clean image for cell counting. Adhesion data for the 1800 s of test are shown in Figure 5. The data show that different materials have distinct adhesion behaviour. A higher bacterial adhesion was observed on the PDMS surface during the 1800 s assay while a lower bacterial adhesion was observed on the PS surface. Additionally, it was observed that bacterial adhesion increases linearly with time. Additional results are shown for PA and PEO in Figure 6 and Figure 7.

**Figure 8:**
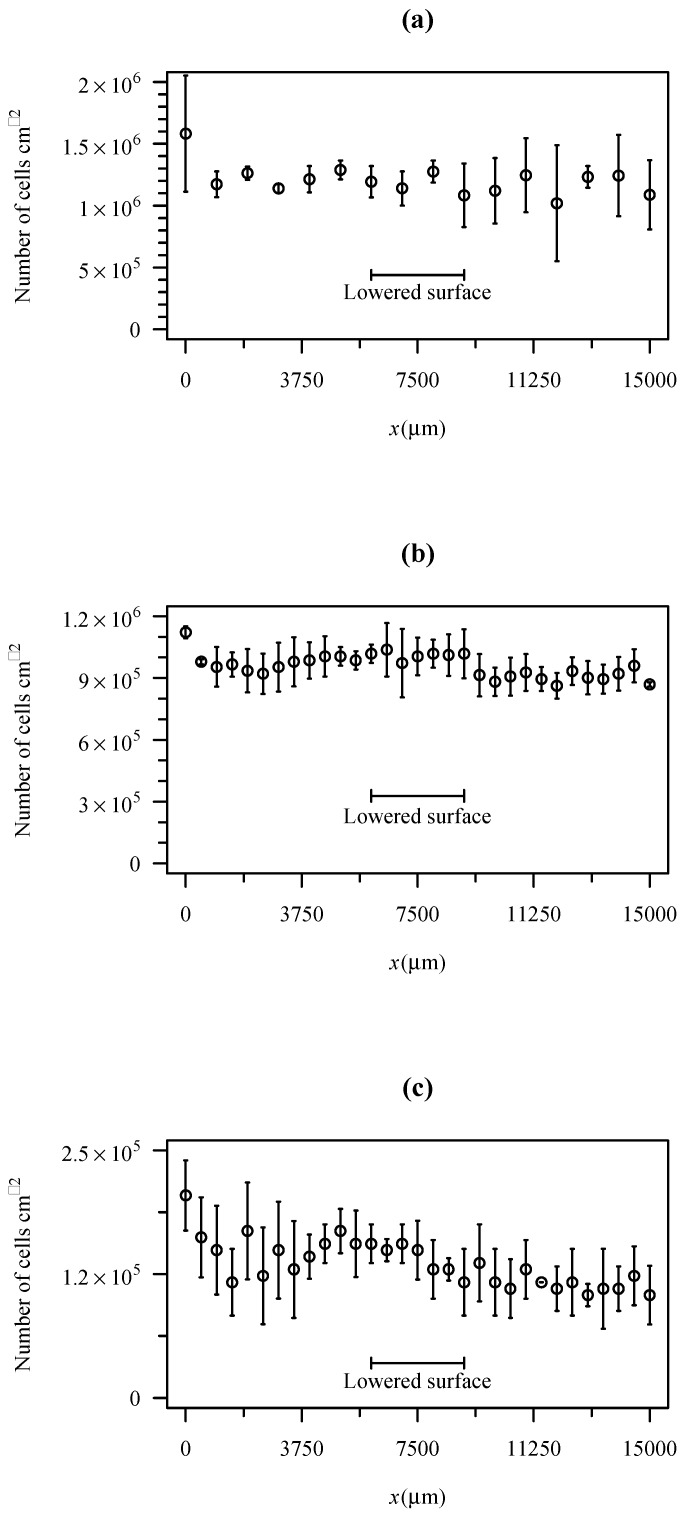
Cell density after 1800 s of adhesion on PDMS for three different nominal wall shear stresses. (**a**) WSS = 0.02 Pa; (**b**) WSS = 0.2 Pa; (**c**) WSS = 1 Pa.

An experiment was performed to test if the unevenness of the channel can change significantly the adhesion rate. To achieve this goal, experiments were performed in a PDMS channel produced by the same fabrication method. First, a layer of PDMS was spincoated over a glass slide. Then, a scotch tape was used to protect a small region, as described in the methods section. Then, a second layer of PDMS was spincoated over the first one. The slide was then used to produce the channels. Figure 8 shows the bacterial cell density along the channel after 1800 s for three different shear stresses. As can be seen in the figure, the presence of a lowered surface in the scotch tape location is not perceptible. The variability of adhesion along the channel is much higher than any possible effect produced by the lowered surface.

### 3.3. Numerical Simulation

**Figure 9:**
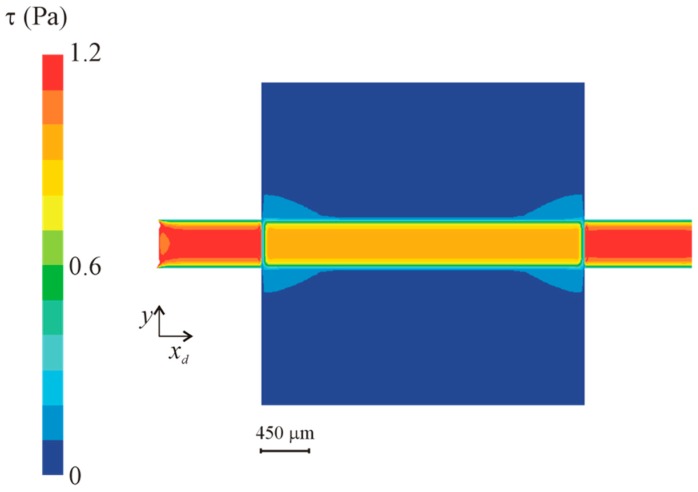
Wall shear stress (WSS) in the lowered surface region for a nominal wall shear stress of 1 Pa.

**Figure 10:**
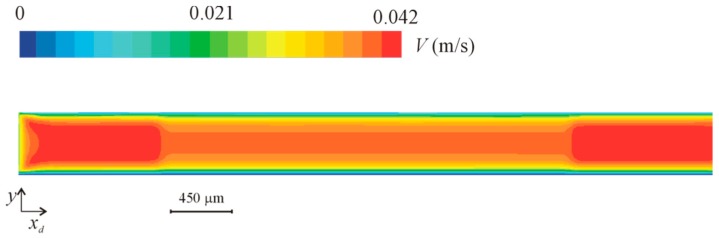
Velocity magnitude in the midplan in the lowered surface region for a nominal wall shear stress of 1 Pa.

Wall shear stress (WSS) at the bottom wall and velocity fields at the midplan are represented in Figure 9 and Figure 10, respectively. These figures show a small velocity decrease in the region of the channel crossing the lowered surface. Lateral regions (see Figure 2) of the lowered surface have an almost zero velocity. The wall shear stress is also smaller in the part of the channel that crosses the lowered surface region, where the region of interest is located.

**Figure 11:**
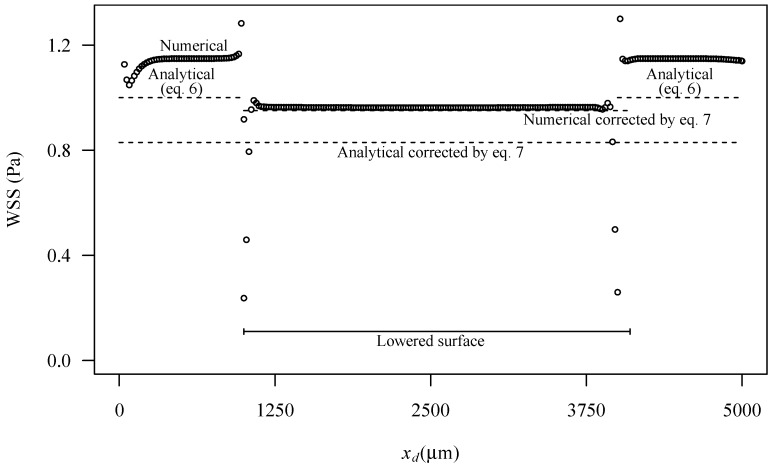
Wall shear stress along the centreline at the bottom surface in the lowered region for a nominal wall shear stress of 1 Pa. Figure shows numerical predictions (symbols), predictions based on analytical Equation (6) and predictions with corrections based on Equation (7). Local domain coordinates are used to represent the distance from the domain inlet.

Figure 11 shows the wall shear stress along the centreline of the bottom wall of the channel. The figure is for the higher nominal WSS studied (1 Pa), but results for the other wall shear stresses, not shown here, are similar. Some edge effects are observable, mainly due to the influence of the inlet and outlet boundary conditions. At the inlet, the edge effects are small, revealing that the flow develops in a short length, and enters fully developed in the lowered surface region. In this lowered region, the wall shear stress is smaller. A small transition region exists of about 300 μm.

The analytical Equation (6) underpredicts the numerical WSS data in the channel outside the lowered region. This result is expected since the analytical equation is exact only for channels with an infinite width. In the present case the ratio between the width and the height of the channel is 4.5, which implies that the velocity is higher than what would be in a channel with infinite width.

The correction—Equation (7)—made on the analytical equation underpredicts the WSS in the lowered surface, while the correction made to the numerical results predicts correctly the WSS in the lowered surface.

## 4. Discussion

A technique developed to create microchannels with polymeric patches for adhesion tests allows the fabrication of patches with several different materials. Surface hydrophobicities of the materials used in this work, range from −65.32 to 0.350 mJ m^−2^ and zeta potential from −11.0 to −29.8 mV. From the bacterial adhesion results on five selected materials (PLLA, PDMS, PA, PEO and PS), it was verified that these different materials led to different adhesion behaviours. According to the thermodynamic theory, a higher bacterial adhesion would be expected on the most hydrophobic surface and a decrease would be expected with decreasing hydrophobicity [45]. A lower bacterial adhesion was in fact obtained in PS, the less hydrophobic surface. However, a higher number of adhered bacteria was observed in PDMS and not in the most hydrophobic surface, PLLA and PEO, which is hydrophilic, has a higher adhesion that PA, which is hydrophobic. Additionally, no correlation between bacterial adhesion and surface charge was found. Although it was verified that bacterial adhesion was not controlled by surface hydrophobicity or charge, other specific parameters of each surface, such as chemical composition, may have affected bacterial adhesion. The effect of surface properties may also be concealed by the flow conditions. The results presented in this paper are for WSS of 0.01–0.02 Pa and low Reynolds number, for which cell sedimentation has relevant contribution to cell adhesion. These results show that different materials, with different properties, were in fact placed in a specific position in the microchannel and induce different bacterial adhesion.

One of the inherent weaknesses of the method is the fact that a region of the microchannel is lowered, i.e., there is a small gap of about 10 μm in the edges of this region. The tests performed with PDMS surfaces (Figure 8) for wall shear stresses between 0.02 Pa and 1 Pa show that the lowered surface does not influence adhesion. Hydrodynamic consequences of this lowered surface were studied in detail by CFD. The CFD study shows that the region has a smaller WSS. Nevertheless, the WSS in this region is stable and calculable by Equation (7). Overall, the results obtained showed that the device performs predictably in adhesion tests and can be adopted for general use in routine tests.

The accuracy of the channels obtained by the current procedure is limited by the accuracy of the underlying microfabrication technique. In the current work the channels were fabricated by xurography, which has an error of approximately 5% to produce channels of 450 μm width [46].

## 5. Conclusions

A new low-cost device for adhesion tests in polymeric surfaces, which can be fabricated in a laboratory with low resources, was developed and characterized. The most expensive equipment used in the fabrication procedure is a spin coater. The fabrication method is suitable to produce microchannels to test bacterial adhesion in different materials, provided that the material is available as a transparent substrate or that the material is a spincoatable transparent polymer that can be casted by solvent evaporation.

The section of the channel containing the region of interest is lowered. However, this lowered surface has a predictable effect on wall shear stress and a negligible one on bacterial adhesion, as shown by adhesion tests and CFD.

The device was validated to be used to perform bacterial adhesion tests under relevant shear stresses. This work contributes to increase the options for experimenters to perform adhesion tests in microfluidic devices, with advantages related to reactant consumption and parallelization.

## Figures and Tables

**Table 1:** Polymers and solvents used to prepare polymeric solutions.

Polymer	Solvent	Polymer Concentration (*w*/*w*)
Polyethylene oxide (PEO)	Dichloromethane (DCM)	1.14%
Poly-l-lactide acid (PLLA)	Dichloromethane (DCM)	5.00%
Polyamide (PA)	Trichloroethanol	0.49%
Polydimethylsiloxane (PDMS)	Curing agent (Sylgard 184)	10.0%

**Table 2:** Hydrodynamic conditions.

Flow Rate (μL/min)	Mean Velocity (m/s)	Reynolds Number	Nominal Wall Shear Stress (Equation (6)) (Pa)
0.667	$2.50\times 10^{-4}$	0.06	0.01
1.35	$5.00\times 10^{-4}$	0.12	0.02
15	$5.56\times 10^{-3}$	1.30	0.2
65.1	$2.41\times 10^{-2}$	5.65	1

**Table 3:** Surface properties of different materials.

Polymer Surface	Hydrophobicity Δ*G* (mJ·m^−2^)	Zeta Potential (mV)
PLLA	−65.32	−27.9
PDMS	−61.82	−29.3
PA	−37.58	−28.0
PEO	0.350	−11.0
PS	−49.56	−29.8
